# Vestibulocochlear implant (VCI) modulation improves mid and high frequency vestibulo-ocular reflex (VOR)

**DOI:** 10.1007/s00415-026-14109-0

**Published:** 2026-09-08

**Authors:** B. L. Vermorken, S. C. J. van Boxel, B. Volpe, N. Guinand, A. Pérez Fornos, E.M.J. Devocht, R. van de Berg

**Affiliations:** 1 Division of Balance Disorders + Mental Health and Neuroscience Research Institute (MHeNs), Department of Otorhinolaryngology and Head and Neck Surgery, Maastricht University, Maastricht, The Netherlands; 2 Division of Otorhinolaryngology Head and Neck Surgery, Department of Clinical Neurosciences, Geneva University Hospitals, Geneva, Switzerland

**Keywords:** Vestibular implant, Modulation, VOR restoration, Bilateral vestibulopathy

## Abstract

**Introduction:**

The vestibulo-ocular reflex (VOR) stabilizes gaze during head movements by generating compensatory eye movements. In bilateral vestibulopathy (BV), loss of vestibular function leads to loss of VOR and consequently, oscillopsia. Currently, no curative treatment exists. Vestibulocochlear implants (VCI) aim to restore vestibular function by modulating electrical stimulation of the ampullary nerves in response to head motion. Although prior studies have demonstrated partial VOR restoration, a comprehensive evaluation across different stimulation conditions remains limited. This study assessed the effects of multiple stimulation conditions on VOR gain in BV patients implanted with the latest VCI prototype.

**Methods:**

Nine BV patients were unilaterally implanted with an intralabyrinthine VCI. VOR responses were assessed using rotatory chair testing (mid-frequency domain) and video head impulse testing (vHIT; high-frequency domain). Five stimulation conditions were evaluated: Reference (VI off), Baseline only (constant stimulation), Default (motion modulation around baseline), Lowered baseline (motion modulation around threshold level), and Boosted stimulation (maximized modulation). Subjects were classified into subgroup A (peak eye velocity, PEV, ≥ 30°/s) during lateral ampullary nerve block stimulation and subgroup B (PEV < 30°/s).

**Results:**

Six subjects were classified in subgroup A. In rotatory chair testing, excitatory VOR gain increased significantly under modulated stimulation at all tested frequencies (*p* < 0.05), with median gains rising from 0.09–0.20 (Reference) to 0.82 (Lowered baseline). No significant inhibitory improvements were observed. In vHIT, subgroup A demonstrated significant excitatory median gain improvements across all canal planes (*p* < 0.005). Lateral canal gains improved from 0.12 (Reference) to 0.69 (Boosted), and anterior canal median gains up to 0.74. Posterior gains improved but remained < 0.60. Inhibitory gains showed no changes. Subgroup B showed no meaningful gain improvements in any condition.

**Discussion:**

Motion-modulated stimulation enhanced excitatory VOR function at mid- and high frequencies in patients who demonstrated higher PEV during block stimulations. However, baseline stimulation alone was ineffective. Lowered baseline and Boosted conditions showed the largest improvements, with gains approaching functional levels in several subjects. However, inhibitory responses remained limited, reflecting physiological and technical constraints. These findings support individualized, frequency-dependent modulation strategies to optimize gaze stabilization and potentially reduce oscillopsia in daily life.

**Supplementary Information:**

The online version contains supplementary material available at https://doi.org/10.1007/s00415-026-14109-0.

## Introduction

Nowadays, cameras use built-in video stabilization to make jitter-free footage. The same applies to our own eyes, stabilizing and reacting to head movements. Every head movement elicits a compensatory eye movement in the opposite direction. This dynamic response is mediated by the vestibular system, primarily through the semicircular canals, and is known as the vestibulo-ocular reflex (VOR). Importantly, the vestibular system exhibits frequency-dependent behaviour. This means that, while at a frequency range between 2 Hz – 10 Hz eye velocity closely matches head velocity (gain close to 1), at both lower and higher frequencies gain decreases [1].

Bilateral vestibulopathy (BV) is defined as a severe chronic loss of vestibular function in both ears. It is a heterogeneous disorder with variable effects on core vestibular functions, resulting in diverse clinical manifestations [2]. Loss of VOR function across mid and high frequencies [3] is typically thought to lead to oscillopsia, one of the hallmark symptoms of BV patients in daily life. This is characterized by blurred vision during head movements. Unfortunately, at this point no curative treatment is available and patients are limited to vestibular rehabilitation programs in order to cope with their symptoms.

The dominant role of the vestibular system’s semicircular canals in gaze stabilization, together with the geometrical configuration, make these structures attractive targets for prosthetic electrical stimulation [4]. The current state of various methodology regarding device designs, surgical and stimulation approaches regarding vestibular stimulation was recently reviewed by [5]. A combined multicanal vestibular (VI) and cochlear implant (CI) research device, also known as the vestibulocochlear implant (VCI), was previously developed by MEDEL and further investigated by the european institute of vestibular implantation (EIVI), also known as the Geneva-Maastricht team. The VCI is designed to restore both vestibular and auditory function in patients with BV and severe sensorineural hearing loss. The latest VCI prototype features three intracanalicular electrodes designed to directly stimulate the ampullary nerves.

A primary goal of VCI stimulation is the artificial restoration of VOR function. This can be achieved by modulating a baseline current delivered to the ampullary nerves, mimicking natural vestibular physiology. Previous studies demonstrated that artificial 1D excitatory vestibular stimulation can evoke bidirectional eye movements and (partially) restore VOR across multiple frequencies. For example, stimulation of the lateral ampullary nerve (LAN) by whole-body rotation improved VOR at 0.5 Hz in three subjects [6]. Moreover, an electrically evoked VOR was observed when all three ampullary nerves were stimulated at frequencies including 0.1, 0.2, 0.5, 1 Hz at 100 ⁰/s in four subjects [7] and 0.5, 1, 2 Hz at 30 ⁰/s peak velocity in seven subjects [8]. Restoration of VOR at high frequency head impulses (5–7 Hz), assessed using the video head impulse test (vHIT), was demonstrated in three subjects [9]. More recently, stimulation parameters were investigated to improve VOR in all directions [10, 11].

Given the wide range and variability of head rotation frequencies and velocities in daily life and the clinical impact of oscillopsia in BV patients, it is crucial to evaluate electrically evoked VOR dynamics comprehensively. The present study aimed to assess the effect of various 3D multicanal motion-modulated stimulation strategies on VOR function in BV subjects implanted with the latest multicanal VCI prototype. By comparing multiple motion-modulation conditions to baseline-only stimulation and a VI OFF condition, this study seeks to provide an initial framework for VOR restoration across different frequency domains. Overall, the results may inform the development of optimized modulation strategies for the multicanal VCI.

## Materials and methods

### Study population

Nine subjects were included in this study. All subjects participated in the VertiGO! Trial, for which the study protocol was published previously [12]. In short, the VertiGO! Trial population consisted of subjects with bilateral vestibulopathy and severe hearing loss in at least the implanted ear. Each subject included in this report was unilaterally implanted with an intralabyrinthine multicanal VCI *(supplied by MED-EL, Innsbruck, Austria*). All three semicircular canals were fenestrated close to the ampulla, to insert one electrode per canal as described previously [13]. Adequate electrode positioning was verified using peri- and postoperative imaging [14, 15].

### Experiments

#### Experimental setup and data collection

To evaluate the effects of different VI stimulation conditions on VOR restoration across multiple frequency domains, all subjects were exposed to two experiments: (1) rotatory chair testing to quantify mid-frequency head-movement responses (see Sect. Rotatory chair), and (2) video Head Impulse Tests (vHIT) to quantify high-frequency responses (see Sect. Video head impulse test). For this study < 0.5 Hz was categorized as low frequency, 0.5–2.0 Hz as mid frequency and > 2.0 Hz as high frequency.

Both assessments were integrated into the VertiGO! trial protocol, as detailed by [12]. The rotatory chair and vHIT experiments were performed once at study inclusion, prior to implantation. These preoperative assessments were conducted to confirm eligibility for bilateral vestibulopathy. The preoperative vHIT measurement was also included in the data analysis. Following implantation, the cochlear implant component was fitted according to standard clinical practice. Afterwards the VI component was fitted as part of the experimental procedures described previously [10]. The outcomes of the short block stimuli presented during the fitting procedure were used to define subgroups of responders as described in Sect. subgroup classification.

After completion of the VI fitting procedure, a reference testing day was conducted to measure vestibular performance in the absence of vestibular stimulation. On this day, the rotatory chair and vHIT experiments were performed once. Subsequently, subjects underwent three separate four-day in-hospital periods of continuous VI stimulation. A three-period crossover design was used with vestibular stimulation condition as the only variable differing between periods. Within each stimulation period, both the rotatory chair and vHIT experiments were administered twice on day 1 and day 3. Rotatory chair and vHIT measurements were always performed directly after each other. Vestibular implant stimulation conditions are described below. The cochlear implant was continuously switched on throughout all study phases and procedures, to facilitate hearing.

#### Rotatory chair

Participants were positioned on an earth-vertical axis rotatory chair (*Ekida GmbH, Buggingen, Germany*) and subjected to horizontal whole-body rotations. These tests were performed in complete darkness. Subjects were instructed to sit still (passively moving along with the chair), look in front of them and keep their eyes open during the trials. The head was 30⁰ pitched forward (without head stabilization) to align the horizontal canal with the horizontal plane. In case the subject could not keep the head stable resulting in severe head bounce, bandage around the forehead and chair was used to keep the head stable. Eye responses were recorded at a sampling rate of 173 Hz with a light-weight 2D video-oculography system (*ICS Impulse device, Natus, Taastrup, Denmark*) with one camera focused on the pupil of the right eye.

Rotational velocities followed a sinusoidal profile with 30°/s peak amplitude [6]. Each measurement consisted of 20 cycles. Input data from the gyroscope and accelerometer of the vestibular implant were recorded to calculate the head velocity. This provided a more precise measure of head movement than chair movement alone, which may be inaccurate when the head was not fully stabilized. Three rotational frequencies were tested: 0.5, 1 and 1.5 Hz. The order of test frequencies was randomized per session. Lower modulation frequencies were excluded based on prior studies indicating a minimal contribution of the natural VOR and a predominant role of the visual system at these frequencies [16]. Consistent with these findings of the natural VOR, electrically evoked VOR responses were negligible as well [6]. Higher rotational frequencies were also excluded to minimize movement artefacts. Despite head stabilization measures, including the use of a bandage, head fixation was insufficient to maintain strict alignment with the rotational motion of the chair, resulting in significant head displacement at higher frequencies.

#### Video head impulse test

vHIT tests were performed according to a previously published experimental setup [17, 18]. In short, subjects were seated on a chair at a distance of 2 m from a white wall. The room was well lit to ensure a small pupil size as required for accurate pupil detection*.* Eye and head velocities were recorded at a sampling rate of 250 Hz with a 2D video-oculography system (*ICS Impulse device, Natus, Taastrup, Denmark*) with one camera focused on the pupil of the right eye. The head band of the goggles was tightly strapped and positioned at least 1 cm away from the external part of the VCI, to exclude any possible interference of the strap with the gyroscopes. The eye position was calibrated using the ICS Impulse two-point calibration [19]. Head impulses were applied by one of two trained examiners (BLV)(BV). The experiment started with impulses in the lateral plane, followed by the left anterior right posterior (LARP) and right anterior left posterior (RALP) planes. Data collection for each plane was considered complete once 20 accepted traces per direction (right/left/up/down) were obtained. A trace was accepted if it met the following criteria: minimum head velocity of 120 ⁰/s, a small amplitude of ± 15°, absence of head bounce exceeding 50% of the peak head velocity, and intact pupil detection.

#### Vestibular implant stimulation conditions

Translation of head movements (velocity, input) into amplitude modulations of the baseline signal (output) was programmed using dedicated research software (*AmpFit, provided by MED-EL, Innsbruck, Austria*). The stimulation profile consisted of charge balanced, cathodic first, biphasic, rectangular pulses, with a specific and continuous rate (varying between subjects, 322–388 pulses per second (see supplementary materials I), an interphase gap of 2.1 µs and phase duration of 200 µs [10, 20].

The individual dynamic range (DR) was determined during the fitting procedure as the range between the threshold of activation (i.e. first measured eye movement response or first perception by participant) and the upper comfortable level (UCL, defined by discomfort or facial nerve stimulation) [21]. The amplitude of baseline stimulation was defined as 50% DR. An overview of the DR width at 50% baseline stimulation per subject and electrode is given in supplementary materials II.

**Fig. 1:**
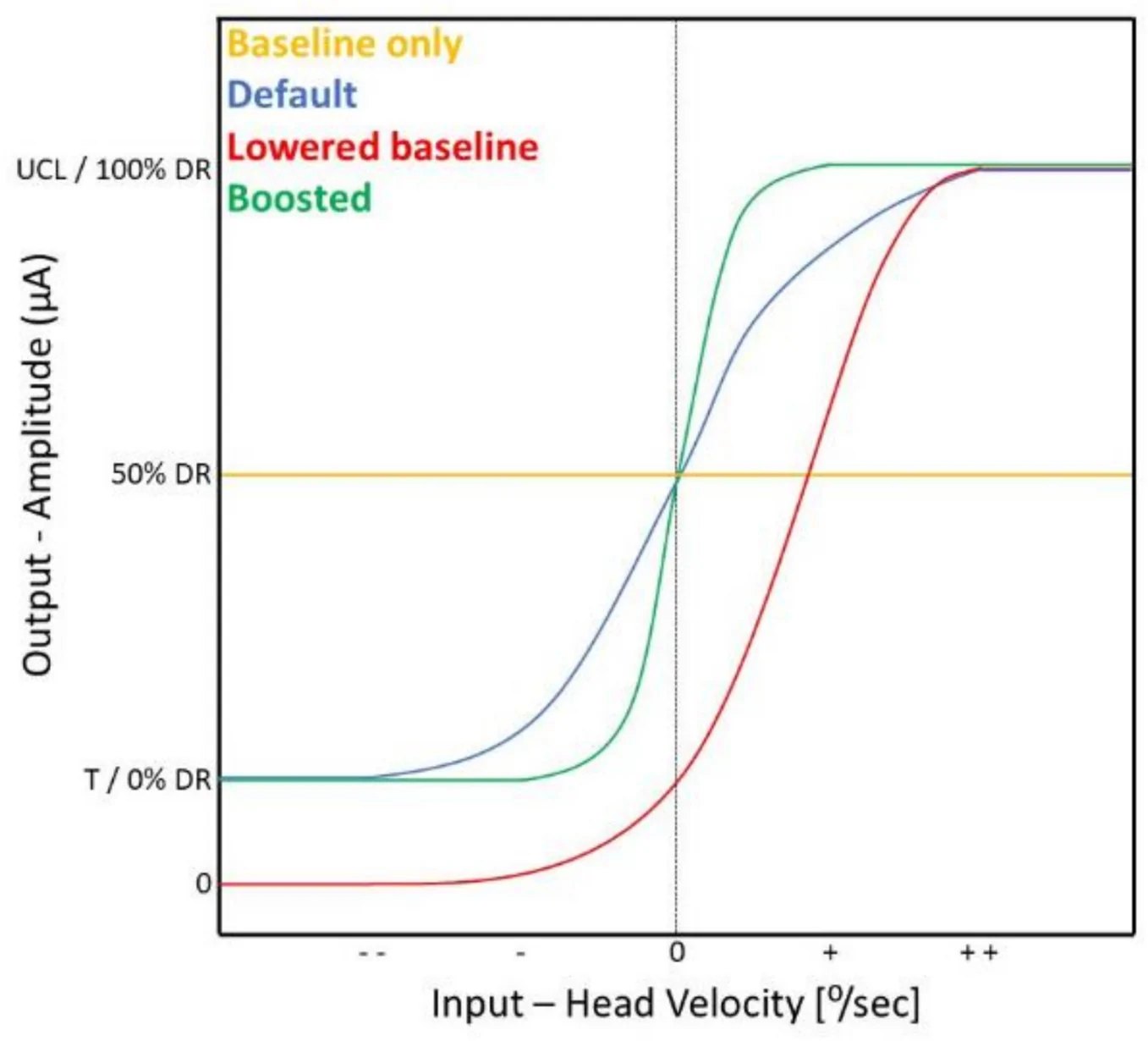
Standardized examples of the stimulation conditions. Three head velocity-to-pulse amplitude sigmoidal mappings (e.g., transfer functions) are shown to illustrate the motion-modulated stimulation conditions. *DR* = Dynamic Range, *UCL* = Upper Comfortable Level, *T* = Threshold

As described earlier, measurements were conducted using multiple stimulation conditions [12]. Table 1 provides an overview of the used conditions. Figure 1 shows standardized examples of the stimulation conditions. In total, five distinct conditions were used in this study. The *Reference* condition was applied during the reference test day, prior to the three periods of continuous VI stimulation. In this condition vestibular stimulation was absent. The *Baseline only*, *Default*, and *Lowered baseline* conditions were randomly assigned to period one, two and three. *Baseline only* consisted of only pulse trains of constant amplitude and rate (baseline 50% DR). Head velocity around each specific SCC axis (input) was used to modulate the amplitude of the stimulation at *Default*, *Lowered baseline* and *Boosted* stimulation. The full dynamic range was used for modulation. At *Lowered baseline* stimulation, the number of current levels for modulation increased (baseline at threshold level). Therefore, it was hypothesized that this condition elicited a larger range of eye velocities. Saturation was kept similar to *Default* stimulation.

**Table 1:** Overview of vestibular stimulation conditions

Condition	Description	Test session
Reference	Vestibular electrode stimulation off	Reference day
Baseline only	Constant-amplitude electrical signal at 50% of the dynamic range to all three vestibular electrodes without modulation	Period 1, 2 or 3
Default	Amplitude modulated stimulation, baseline stimulation at 50% of the dynamic range to all three vestibular electrodes	Period 1, 2 or 3
Lowered baseline	Amplitude modulated stimulation, baseline stimulation at the subject’s threshold to all three vestibular electrodes	Period 1, 2 or 3
Boosted*	Motion-modulated stimulation using maximized modulated amplitudes, baseline stimulation at 50% of the dynamic range to all three vestibular electrodes	End of period 3

Cochlear stimulation was activated in all conditions* Boosted stimulation was only applied for additional vHIT experiment in subjects showing peak eye velocities ≥ 30 ⁰/s. This was not performed in rotatory chair experiments

The head velocity-to-pulse amplitude sigmoidal mappings can be fitted with varying degrees of steepness. As previously published, PEV was strongly correlated with stimulation amplitude [22–24]. A steeper mapping increases current delivery faster in response to head movements, which could significantly influence the maximum PEV. The *Boosted* condition was evaluated at the end of period three in those subjects who demonstrated PEV ≥ 30⁰/s during the block pulses in the fitting experiments (see Sect. subgroup classification). A steeper sigmoid function was applied (baseline 50% DR), where stimulation currents increase faster for a given head velocity. It was expected that in these subjects, further improvement of gain restoration using this additional strategy could be possible. This extra condition was only applied for the vHIT experiment, not in rotatory chair experiments to prevent discomfort associated with a longer-duration protocol.

#### Subgroup classification by PEV

Because peak eye velocity (PEV) during brief vestibular stimulation was hypothesized to play an important role in VOR restoration, data from the VI fitting procedure were used as outline for a predefined subgroup analysis. During the extensive preceding VI fitting procedure [10], PEV was measured during 2-s block stimuli selectively given to each ampullary nerve (LAN, SAN, PAN separately), in which the amplitude increased step by step from baseline level to the UCL. A PEV ≥ 30°/s during an excitatory 2-s block stimulation to UCL at LAN, SAN or PAN, was classified as indicative of sufficient VOR restoration (high responder), defining subgroup A. A PEV < 30°/s was classified as indicative of inadequate restoration (low responder), classified as subgroup B. This cut-off was chosen, because a peak angular velocity of 30°/s was given to the chair in the rotatory chair experiments [25, 26].

### Data processing

#### Rotatory chair

Head and eye velocity traces were imported and cross-correlated in a dedicated eye movement analysis software (*custom eye analysis research software provided by MED-EL, Innsbruck, Austria*). Only artifact-free cycles were included in the analysis. Segments exceeding eye acceleration > 1000°/s^2^ were deleted, since these represented blinks or other artefacts. There was no replacement of deleted segments by interpolated values.

PEV and peak head velocity were determined by fitting half-sinusoidal functions to the filtered response traces, as these phases may exhibit asymmetry [26]. The half-sinusoid corresponding to movement toward the implanted side was defined as the excitatory phase, while the half-sinusoid away from the implanted side was defined as the inhibitory phase. 

Excitatory and inhibitory VOR gain values were obtained for each cycle by dividing PEV by peak head velocity. For each condition and per frequency, mean gain was calculated from the set of accepted cycles using the first 15 accepted cycles.

#### Video head impulse test

Head and eye velocity traces were exported and further processed using a custom-made software written in Python v.3.10., as described previously [27]. After automated processing, traces were manually inspected and removed if artefacts were present, based on the methodology of Mantokoudis et al. [28]. Saccades were evaluated and included if the saccade matched a minimum positive amplitude of 60 ⁰/s, a minimum duration of 150 ms and occurred after head impulse onset with a maximum latency of 600 ms. Negative saccades were excluded from further analysis. A trace with nystagmus, identified as multiple large eye responses with fixed time windows, was manually removed when visually detected. If, based on the properties of the trace, no clear saccadic response could be identified, visual inspection of the recorderd eye video was done to decide final inclusion or exclusion.

VOR gain values were calculated by dividing the area under the curve of the desaccaded eye velocity trace with the area under the curve of the head velocity trace, over the interval between head onset and offset [27]. No interpolation was applied. The mean VOR was calculated from the first 10 artefact-free traces per plane and per head impulse direction: excitatory and inhibitory phase, comparable to the rotatory chair.

### Statistical analysis

Rotations towards the direction of the implant side (excitatory rotations) were analysed separately from movements away from the implant side (inhibitory rotations). The median of the VOR gain values was calculated per subject, condition and subsequently compared across the five stimulation conditions (see Sect. Vestibular implant stimulation conditions) within each plane, frequency and direction. Analyses were performed separately for the two subgroups (A vs B, see Sect. subgroup classification). For the stimulation weeks, it was expected that the stimulation effect would be most pronounced on day 3. Therefore, only data collected on day 3 of each week were included in the statistical analyses. No test–retest statistics were used between day 1 and day 3 because the stimulation duration differed between the two measurement sessions. 

In rotatory chair experiments (horizontal plane), gain comparisons were made per frequency, 0.5 1.0 and 1.5. In the vHIT experiment, gain comparisons were made per plane, lateral/LARP/RALP planes. Raw traces were visually interpreted, before statistical analysis of gain values.

Given the small sample size and the expected non-normal distribution of the data, non-parametric statistical methods were used. To assess differences in VOR gain across stimulation conditions for each experiment (per subgroup, plane, frequency and direction), the Friedman test was applied. When the Friedman test indicated a significant overall effect (alfa < 0.05), post-hoc pairwise comparisons were conducted using the Wilcoxon signed-rank test, with Bonferroni-adjusted p-values to control for multiple comparisons. VOR gains were presented as the median per subgroup, based on the mean VOR gain values per subject.

## Results

### Collected data

#### Included subjects

Table 2 provides an overview of the included subjects, including the preoperative VOR gain values collected with rotatory chair (0.1 Hz) and vHIT at inclusion. A notable residual function was observed in the anterior canals for VCI-5, and in the horizontal and posterior canals for VCI-6. Yet, VCI-5 showed severe loss (< 0.6) in the other semicircular canal planes and VCI-6 showed a sum of bithermal maximum peak velocity on each side of 3°, bilateral VOR gain in other planes < 0.7 and rotatory chair gain of 0.16. Therefore, both subjects meeting all minor implantation criteria [29].

**Table 2:** Detailed overview of included subjects

ID	Etiology	Age (yrs)	Symptom duration (yrs)	Implantation side	Rotatory chair (0.1Hz)	Pre-operative VOR gain Video Head Impulse test					
						Lateral right	Lateral left	Anterior right	Anterior left	Posterior right	Posterior left
VCI-1	DFNA-9	54	7	Right	0.12	0.19	0.32	0.40	0.59	0.33	0.39
VCI-2	Auto-immune	65	21	Right	0.04	0.08	0.32	0.23	0.10	0.08	0.04
VCI-3	DFNA-9	52	30	Left	0.01	0.14	0.30	0.35	0.51	0.06	0.26
VCI-4	DFNA-9	66	10	Right	0.03	0.07	0.20	0.06	0.27	0.11	0.29
VCI-5	Idiopathic	28	4	Right	0.07	0.39	0.49	0.69	0.84	0.51	0.25
VCI-6	Meniere’s Disease	66	25	Right	0.16	0.81	0.90	0.49	0.39	0.61	0.62
VCI-7	DFNA-9	62	6	Left	0.02	0.10	0.20	0.23	0.36	0.30	0.15
VCI-8	Skull base fracture	63	< 1	Right	0.01	0.05	0.03	0.11	0.07	0.04	0.05
VCI-9	Skull base fracture	62	< 1	Right	0.01	0.02	0.03	0.05	0.04	0.02	0.01

#### Subgroup classification

**Fig. 2:**
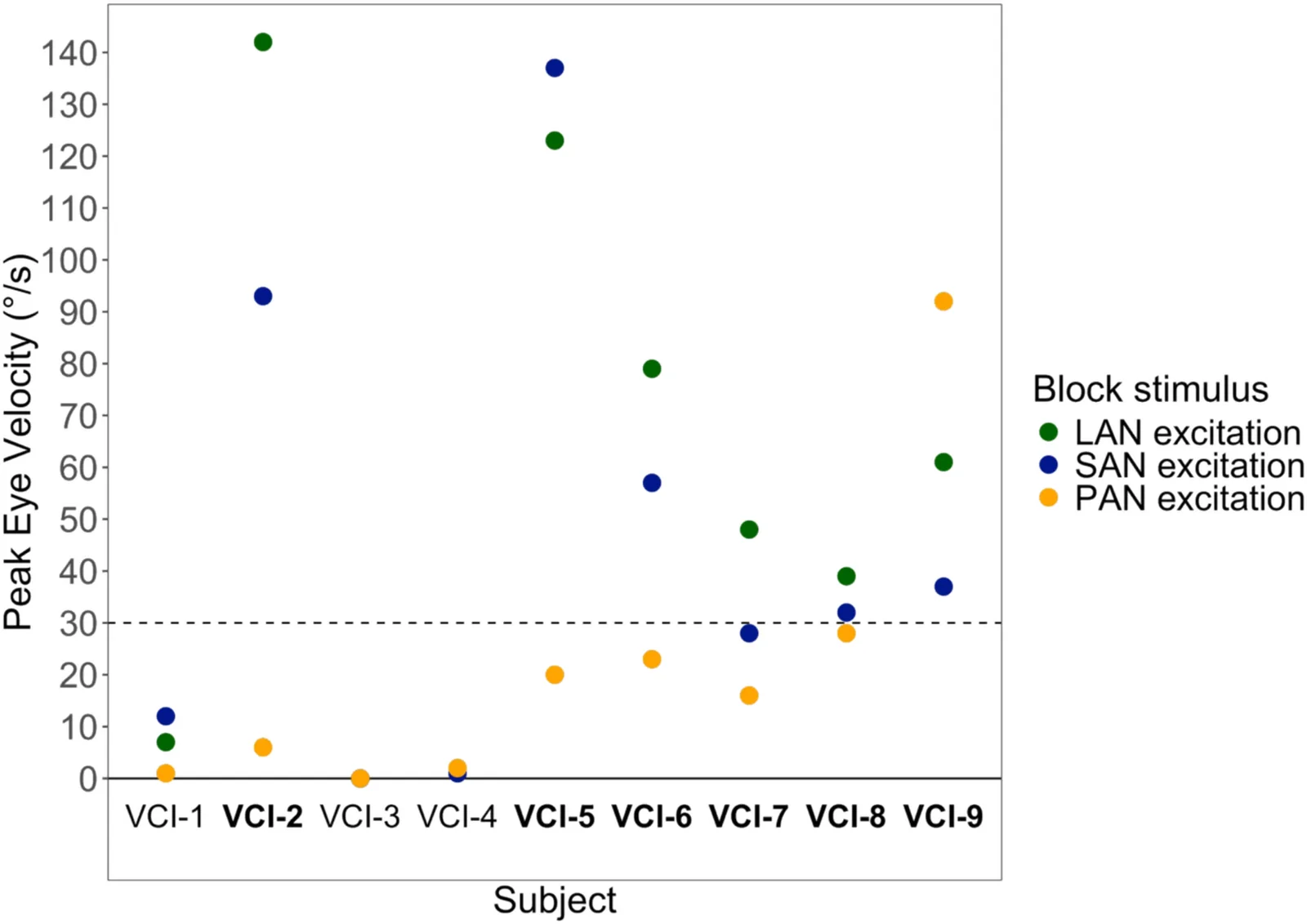
Peak Eye Velocities recorded during an excitatory 2-s block stimulus from baseline level to the upper comfortable level (UCL). The dashed line represents the classification threshold of 30 ⁰/s. Subgroup A subjects are displayed in bold. *LAN* = lateral ampullary nerve, *SAN* = superior ampullary nerve, *PAN* = posterior ampullary nerve

Each subject was classified to subgroup A or B for further analysis. Figure 2 presents PEVs during excitatory block stimulations per stimulating electrode (LAN, SAN, PAN). Six subjects showed PEVs ≥ 30°/s for LAN or SAN stimulations and were classified in subgroup A: VCI-2, VCI-5, VCI-6, VCI-7, VCI-8, VCI-9. The other subjects were classified in subgroup B. PAN stimulation showed in general lower PEVs, except for VCI-9.

### Rotatory chair test

#### Qualitative characteristics

**Fig. 3:**
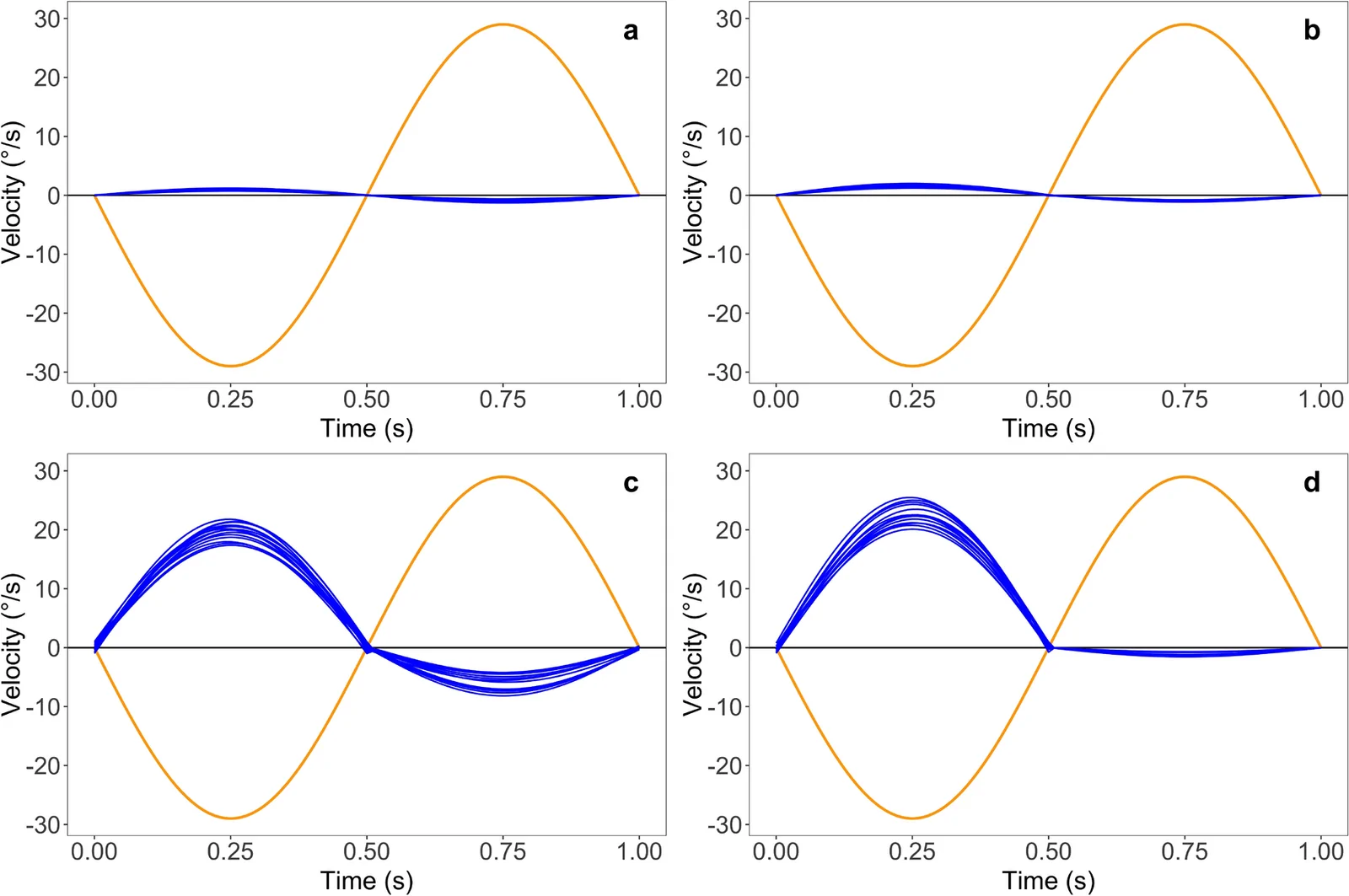
Representative averaged traces from the 1 Hz sinusoid rotatory chair measurements, for all tested VI stimulation conditions in subject VCI-9 (group A). **a** Reference, **b** Baseline only, **c** Default, **d** Lowered baseline. Head traces displayed in orange; eye traces displayed in blue

The overall pattern of the observed data within subgroup A was comparable between subjects and frequencies. Figure 3 presents averaged traces from 1 Hz sinusoidal rotatory chair measurements across all VI stimulation conditions in subject VCI-9. This example is broadly representative for subgroup A. Orange traces indicate negative half-sinusoid fits for excitatory head rotations and positive half-sinusoid fits for inhibitory head rotations, while the corresponding eye movement traces are shown in blue. In this example, *Baseline* stimulation (b) was not associated with observable changes in eye movement responses. By comparison, *Default* stimulation (c) and *Lowered baseline* (d) demonstrated a clear ability to restore eye movement responses during excitatory head rotations. Although *Default* stimulation produced limited eye movement responses during inhibitory head rotations, no clinically relevant responses (eye movement responses showed < 30% of the head velocity) were observed under inhibition. Subgroup B did not show significant eye movement responses. A representative example (VCI-1) of the data collected in subgroup B can be found in supplementary materials III.

#### VOR gain

**Fig. 4:**
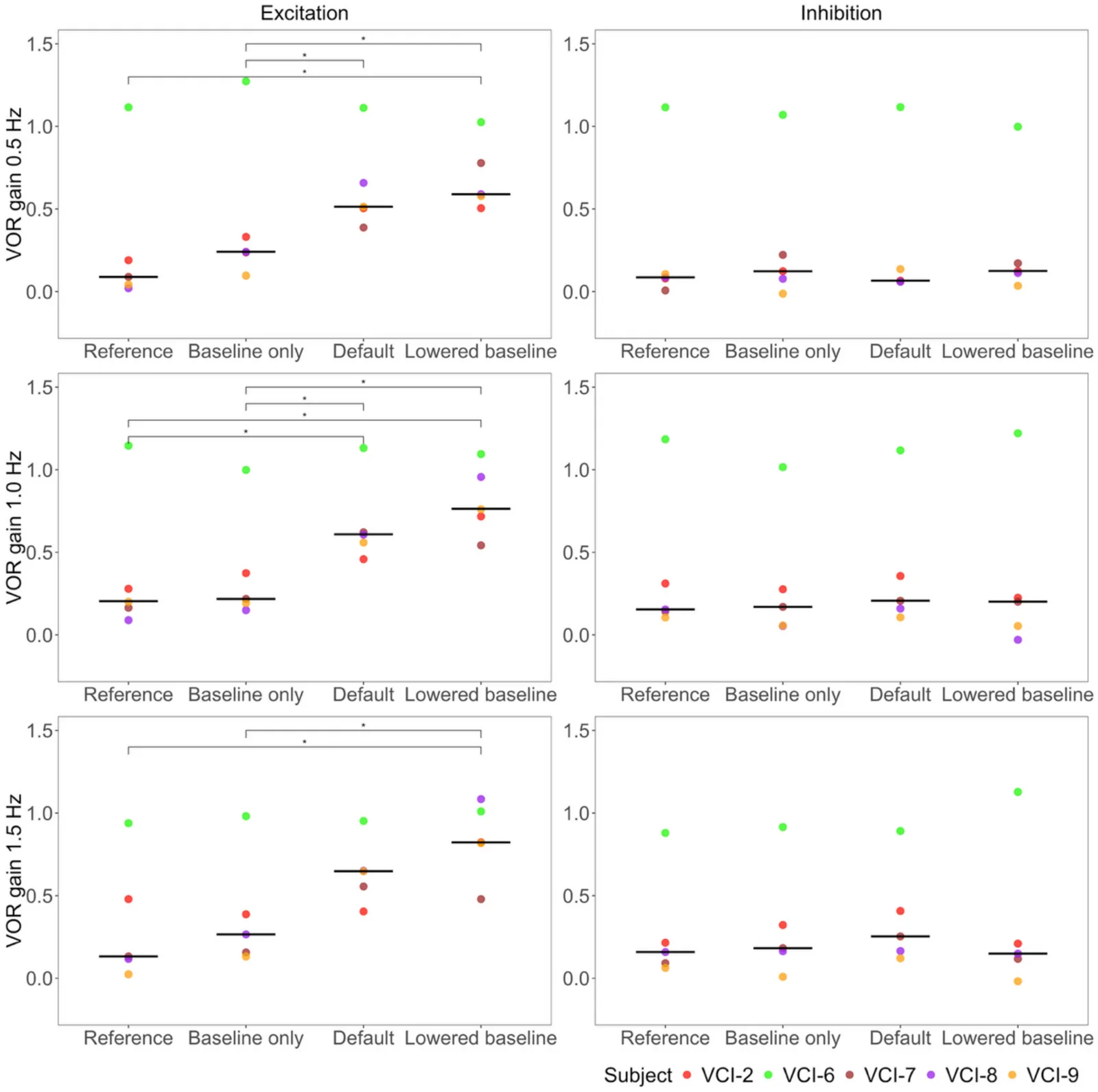
Overview of the median of mid-frequency VOR gain per stimulation condition, frequency and direction for subgroup A. * *p* < 0.05.VCI-5 was excluded due to hardware-related noise, VCI-6 is shown but excluded for further analysis as outlier

An overview of the median VOR gain per stimulation condition, frequency and direction for subgroup A is presented in Fig. 4. Data from subject VCI-5 were excluded due to hardware-related noise, resulting in traces unsuitable for analysis. VOR gain values for subject VCI-6 were consistently elevated across all tested frequencies during both excitatory and inhibitory rotations, marking them as outliers (green markers in Fig. 4). As presented in Table 2, VCI-6 showed bilateral residual function in the lateral plane. Consequently, this subject was excluded from the statistical analysis. As a result, subgroup A comprised four subjects.

Analysis of subgroup A revealed significant differences in median VOR gain between stimulation conditions during excitatory rotations at 0.5 Hz (χ^2^(3) = 11.1, *p* = 0.011), 1.0 Hz (χ^2^(3) = 10.2, *p* = 0.016) and 1.5 Hz (χ^2^(3) = 8.1, *p* = 0.043).

Post hoc analyses revealed significant differences in VOR gain between stimulation conditions within each of the three frequencies during excitatory rotations. At 0.5 Hz, *Reference* condition showed a median VOR gain of 0.09, which increased significantly to 0.59 in the *Lowered baseline* condition. *Lowered baseline* stimulation (0.59) also showed a significant improvement in median VOR gain compared to *Baseline only* (0.24). *Default* stimulation (0.51) similarly demonstrated a significant increase in median VOR gain relative to *Baseline only* (0.24).

At 1 Hz, *Default* (0.61) and *Lowered baseline* (0.76) stimulation showed significantly higher median VOR gain values compared to *Reference (0.20)* and *Baseline only* stimulation (0.22). 

At 1.5 Hz, *Lowered baseline* stimulation (0.82) resulted in a significant increase in VOR gain compared to *Reference* stimulation (0.13) and *Baseline only* (0.27). 

Inhibitory rotations did not result in statistically significant differences at 0.5 Hz (χ^2^(3) = 2.1, *p* = 0.551), 1.0 Hz (χ^2^(3) = 5.4, *p* = 0.144) or 1.5 Hz (χ^2^(3) = 5.7, *p* = 0.127). However, on subject level, VOR gain improvement was observed during *Default* and *Lowered baseline* condition.

An overview of the median mid-frequency VOR gain data for subgroup B is provided in supplementary materials IV. Consistently low VOR gains (≤ 0.40) in this subgroup were observed, with no evidence of improvement under any stimulation condition during either excitatory or inhibitory stimulation. This pattern was observed across all tested frequencies (0.5, 1.0, and 1.5 Hz).

### Video head impulse test

#### Qualitative characteristics

##### Excitation

**Fig. 5:**
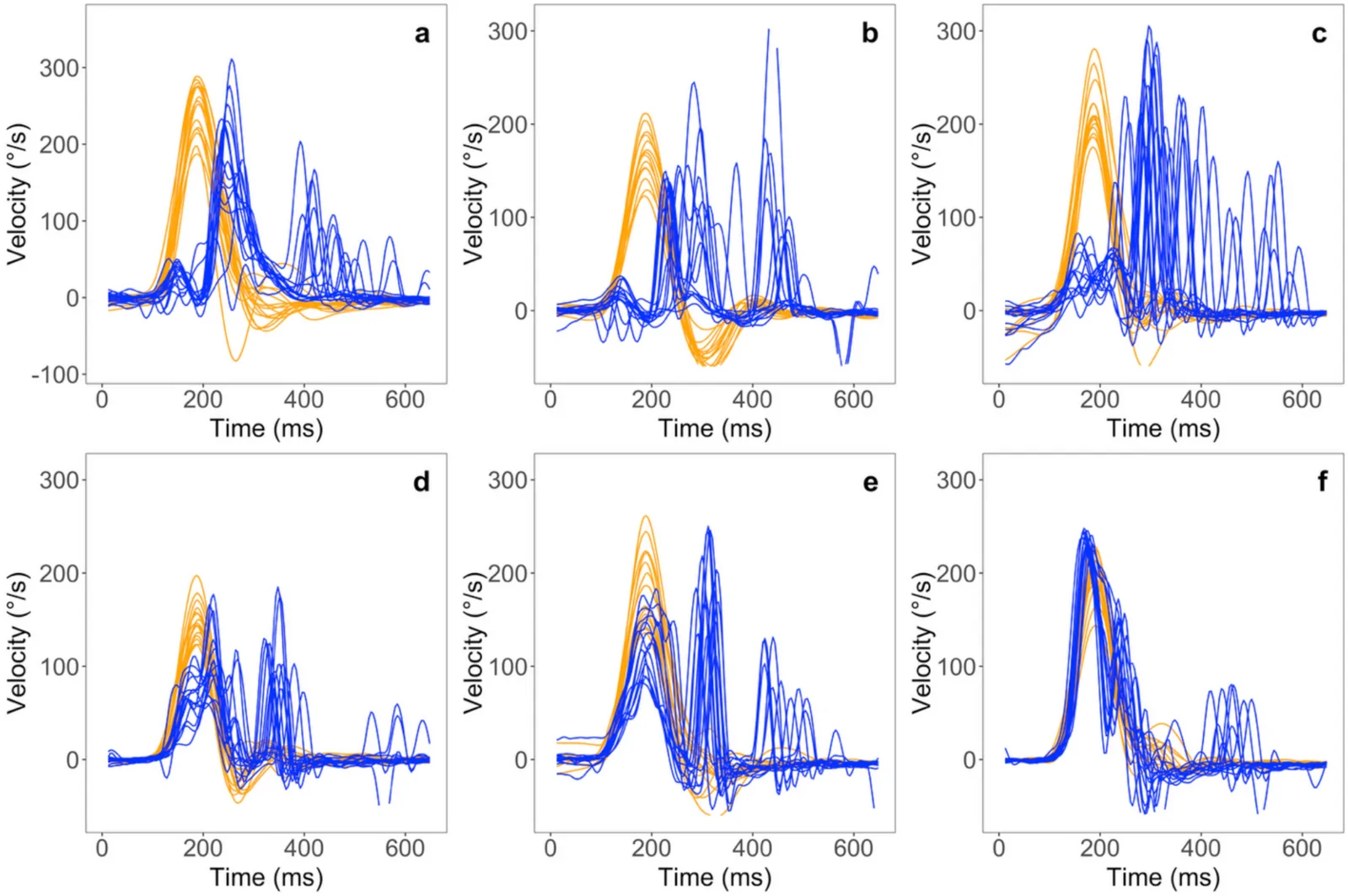
Representative pooled traces from the lateral, excitatory, video Head Impulse Tests for all stimulation conditions in subject VCI-6. **a** Preoperative, **b** Reference, **c** Baseline only, **d** Default, **e** Lowered baseline, **f** Boosted. Head traces displayed in orange, eye traces displayed in blue

The overall pattern of the observed data for excitatory head impulses within subgroup A was comparable between subjects and planes. As example, Fig. 5 shows raw vHIT traces across all VI stimulation conditions in subject VCI-6 in the lateral plane. In the *Reference* test (a), a high frequency of both overt and covert saccades and a low VOR gain was observed. A similar low VOR gain and saccadic pattern were evident during *Baseline* stimulation (b). Under *Default* stimulation (c), eye movement responses during the head impulse increased, resulting in improvement of the gain. This was accompanied by changes in saccadic responses: these occurred less frequently, with shorter latencies, and in a more organized pattern. Further improvement of the eye movement traces was observed under *Lowered baseline* (d) and *Boosted* (e) conditions, characterized by increased peak amplitudes and a more bell-shaped profile. A slight delay in the onset of eye movement responses was present across most conditions, only *Boosted* modulation resulted in improved onset timing. However, the peak of eye velocity did not fully match with the amplitude and timing of the peak of head velocity during the *Boosted* condition (e).

**Fig. 6:**
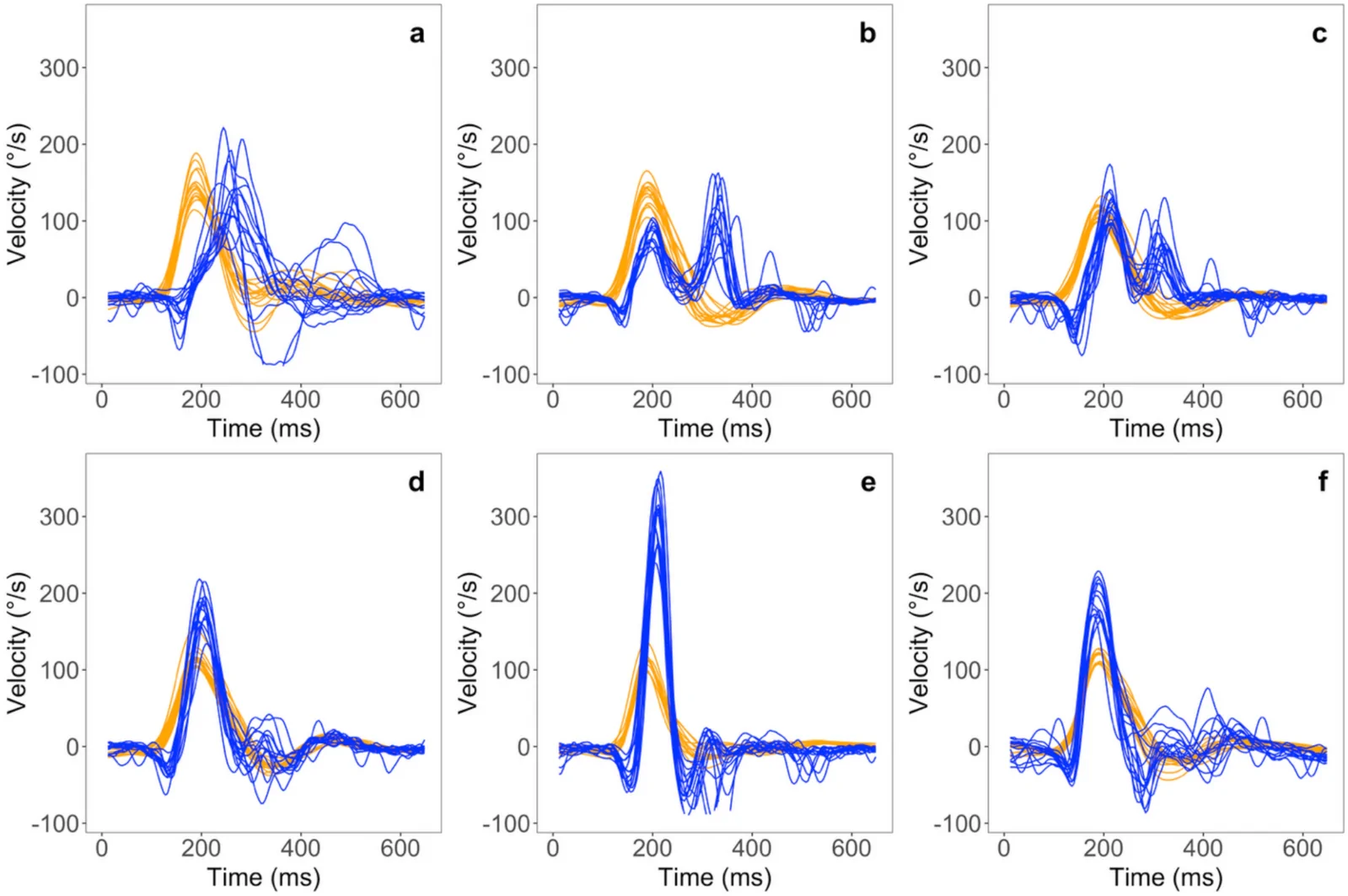
Representative pooled traces from the anterior, excitatory, video Head Impulse Tests for all stimulation conditions in subject VCI-5. **a** Preoperative, **b** Reference, **c** Baseline only, **d** Default, **e** Lowered baseline, **f** Boosted. Head traces displayed in orange, eye traces displayed in blue

While Fig. 5 displays an illustrative example of most data from subgroup A, subject VCI-5 showed a notable deviation from this pattern. Excitatory rotations in the anterior plane in this subject produced a distinct vHIT response pattern. Subject VCI-5 happened to show bilateral residual function during the preoperative measurements in this plane. Figure 6 shows the raw vHIT traces for each stimulation condition in the anterior plane. Overt saccades were observed during the *Reference* (a) and *Baseline only* (b) conditions. These were more organized than those shown in Fig. 5 and were largely absent under modulation conditions (c-e). With modulation, VOR gain increased beyond 1.0, resulting in an overcompensated eye response and a hyperfunctional gain, most prominently during the *Lowered baseline* (d) condition. Additionally, the *Boosted* condition (e) reduced the delay of the eye movement response onset. *Boosted* stimulation resulted in less overcompensation.

Subgroup B did not show significant changes in eye movements between stimulation conditions resulting in VOR improvement. A representative example (VCI-1) of excitatory lateral vHIT traces from subgroup B is provided in supplementary materials V.

##### Inhibition

**Fig. 7:**
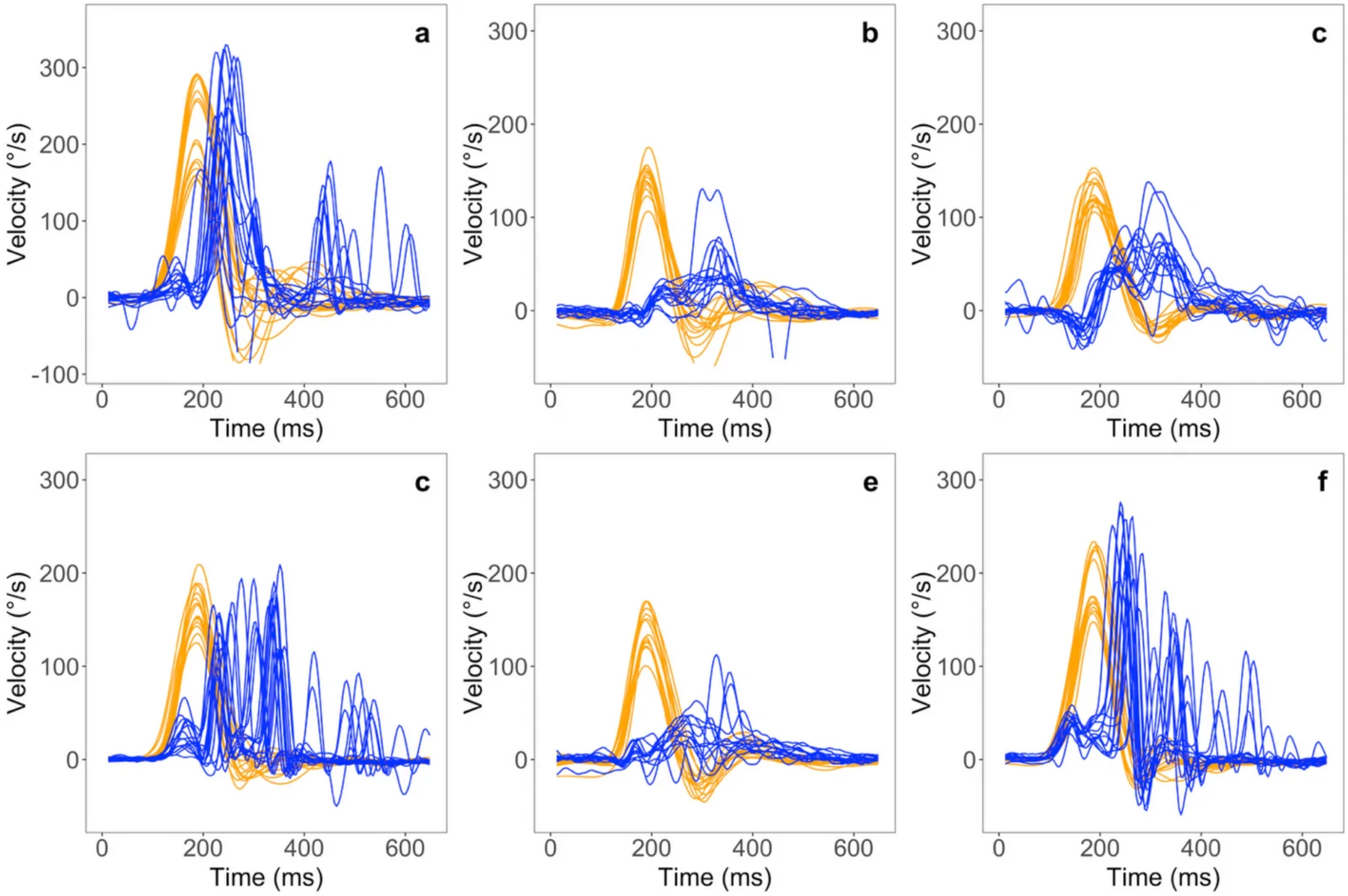
Representative pooled traces from the lateral, inhibitory, video Head Impulse Tests for all stimulation conditions in subject VCI-6. **a** Preoperative, **b** Reference, **c** Baseline only, **d** Default, **e** Lowered baseline, **f** Boosted. Head traces displayed in orange, eye traces displayed in blue

Inhibitory head impulses were again comparable across subjects and planes within subgroup A. Figure 7 shows raw vHIT traces across all VI stimulation conditions in subject VCI-6 during inhibitory head impulses in the lateral plane. The eye movement responses lacked sufficient velocity to produce an improvement in gain in any of the stimulation conditions. Saccadic activity was observed with increased latency with *Reference* (a), *Baseline only* (b) and *Lowered baseline* (d) stimulation (not visible in Fig. 7). *Default* (c) and *Boosted* (e) stimulation elicited only small eye movements and shorter latencies in the saccadic activity.

Inhibitory traces in subgroup B showed no significant changes in eye movement responses across conditions and were therefore comparable to the excitatory traces showed in supplementary materials V.

#### VOR gain

**Fig. 8:**
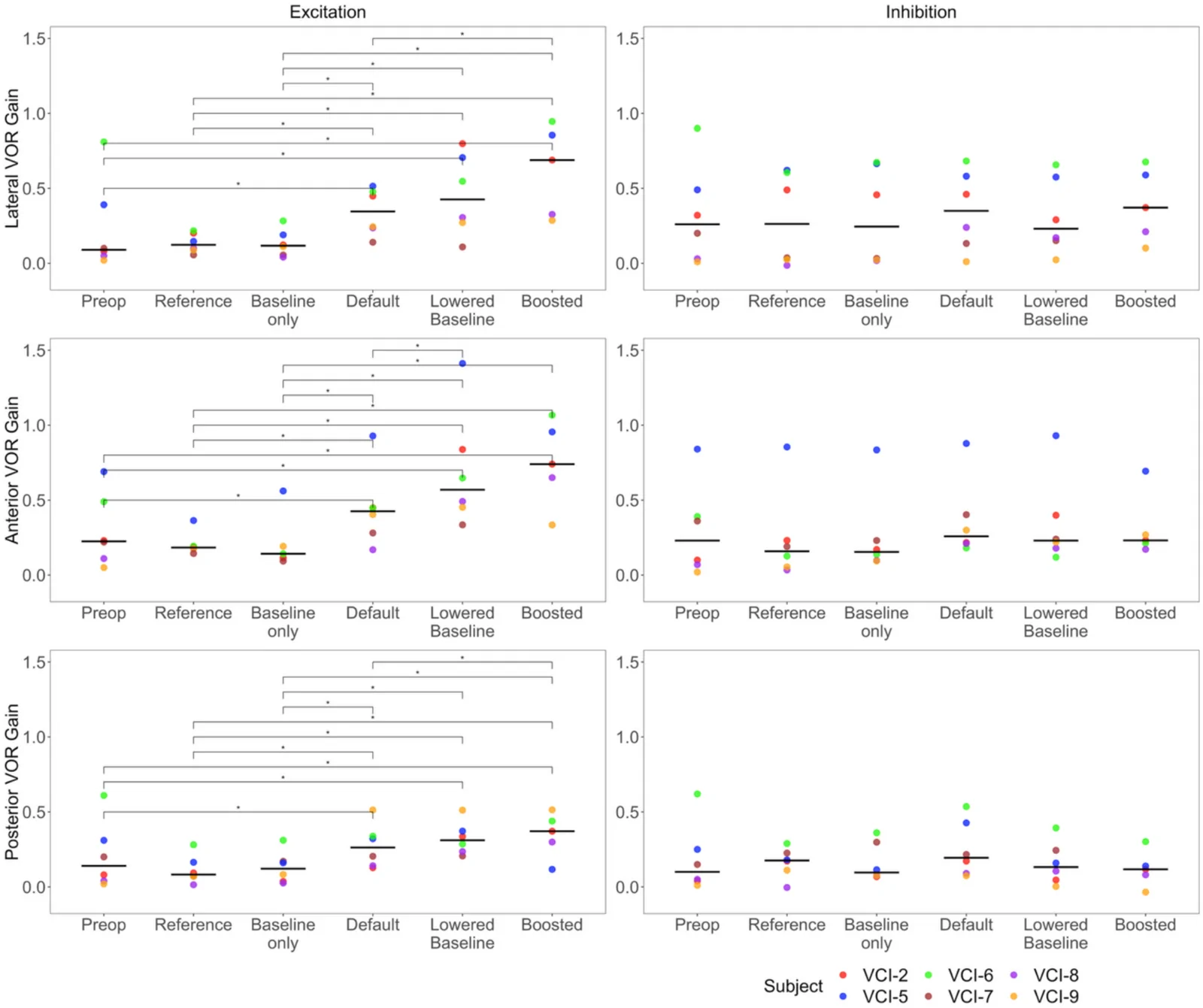
Overview of the median of high-frequency VOR gain per stimulation condition and direction for subgroup A. * *p* < 0.05. Preoperative VOR gain values measured at inclusion (Table 2) were included in this Figure (Preop)

An overview of the median VOR gain per stimulation condition, plane and direction for subgroup A is presented in Fig. 8 No *Boosted* condition was applied in subject VCI-7 due to technical reasons, this subject was deleted from the statistical comparisons.

Analysis of subgroup A revealed significant differences in VOR gain between stimulation conditions during excitatory impulses in the lateral plane (χ^2^(4) = 18.4, *p* = 0.001), anterior plane (χ^2^(4) = 14.88, *p* = 0.004) and posterior plane (χ^2^(4) = 17.12, *p* = 0.002). Post hoc analysis revealed significant improvements in VOR gain across stimulation conditions in all semicircular canal planes for excitatory rotations.

In the lateral plane, the preoperative median gain was 0.09, the *Reference* condition showed a median VOR gain of 0.12 and *Baseline-only* resulted in a median VOR gain of 0.11. A significant increase in median VOR gain was observed with *Default* stimulation (0.34), *Lowered Baseline* (0.43) and *Boosted* (0.69) conditions. Moreover, the median VOR gain in *Boosted* condition was significantly higher compared to *Default* stimulation.

In the anterior plane, the preoperative median gain was 0.22, *Reference* condition resulted in a median VOR gain of 0.18 and *Baseline-only* 0.12. *Default* stimulation (0.42), *Lowered Baseline* (0.57) and *Boosted* (0.74) conditions showed a significant increase in median VOR gain. In this plane, the median VOR gain in *Lowered Baseline* condition was also significantly higher compared to *Default* stimulation.

In the posterior plane, the preoperative median gain was 0.14, *Reference* condition demonstrated a VOR gain of 0.08 and *Baseline-only* condition 0.13. *Default* stimulation (0.26), *Lowered Baseline* (0.31) and *Boosted* (0.40) conditions showed a significant increase in median VOR gain. Comparable to the lateral plane, median VOR gain in the *Boosted* condition was significantly higher compared to *Default* stimulation.

Inhibitory impulses did not result in significant differences between stimulation conditions in the lateral plane (χ^2^(4) = 3.5, *p* = 0.475), anterior plane (χ^2^(4) = 6.59, *p* = 0.159) or posterior plane (χ^2^(4) = 5.45, *p* = 0.24).

An overview of the mean VOR gain data for subgroup B is provided in supplementary materials VI. Analysis of this subgroup revealed consistently low VOR gains (≤ 0.40), with no evidence of improvement under any stimulation condition during either excitatory or inhibitory stimulation. This pattern was observed across all tested planes (lateral, anterior, posterior).

## Discussion

### Overview

Excitatory motion-modulated vestibular stimulation enhanced both mid- and high-frequency VOR gains in subjects who showed substantial eye movement responses in the lateral plane to 2-s block stimuli (PEV > 30°/s; subgroup A), whereas subjects with minimal eye movement responses (PEV < 30°/s; subgroup B) showed no significant VOR changes under any condition. Moreover, results showed that the *Baseline only* condition was not sufficient to improve VOR in mid- and high-frequency rotations compared to the reference (VI-OFF) condition. Mid-frequency VOR during excitatory rotations increased significantly by using *Lowered baseline* stimulation in all tested frequencies, *Default* stimulation increased VOR gain at 0.5 and 1.0 Hz. The magnitude of improvement followed a consistent pattern, with the smallest increase observed in the *Default* condition, a larger increase with the *Lowered baseline* condition, and the greatest improvement with *Boosted* stimulation. This trend was consistent across all three planes when compared with unmodulated stimulation and preoperative measurements. Inhibitory motion-modulated vestibular stimulation did not show any statistically significant changes in both mid- and high-frequency VOR gain at group level.

### Rotatory chair

Four subjects from subgroup A were eligible for the rotatory chair analysis. Modulated stimulation increased VOR gain during excitatory rotations at all three tested frequencies. Both the *Default* and *Lowered baseline* conditions produced significantly higher gains compared with the *Reference* and *Baseline only* conditions. Although the *Lowered baseline* condition was expected to further enhance excitatory responses by increasing the dynamic range available for excitation, gains were not statistically different from those observed with *Default* stimulation. Nevertheless, all subjects except VCI-7 demonstrated higher VOR gain with *Lowered baseline* stimulation, suggesting a potential trend that may achieve statistical significance in a larger cohort with greater statistical power.

The limited effects of inhibitory vestibular stimulation are consistent with previous observations [24] (*Ten Hoor *et al.,* manuscript in preparation)*. Consistent with these findings, a reduced contribution of inhibitory inputs to self-motion perception was recently observed [20]. In Fig. 3, presenting the results of VCI-9, the adverse effect of *Lowered baseline* stimulation during inhibitory rotations is visible, as the minimal stimulation effect of *Default* stimulation on inhibition disappears in this subject. As shown in Fig. 4, subjects did not demonstrate measurable changes during inhibitory rotations.

Although VCI-6 fulfilled the general implantation criteria [29] and showed insufficient VOR gain of 0.16 at inclusion during the rotatory chair test at 0.1 Hz, this subject demonstrated residual function (> 0.7) of the high frequency VOR gain in the lateral plane measured with vHIT preoperatively. Postoperatively, this residual function was also reflected in the mid-frequency VOR responses (0.5–1.5 Hz). The presence of VOR responses during both excitatory and inhibitory stimulation is most likely explained by compensation from the contralateral non-implanted side, which can provide sufficient vestibular input following long-term vestibular loss. In contrast, preserved function of the implanted side is considered highly unlikely because the implanted electrodes mechanically obstruct (i.e. plug) the semicircular canals, thereby preventing normal endolymph flow.

### Video Head Impulse Test

In vHIT, all three modulation paradigms (*Default*, *Lowered baseline*, and *Boosted*) significantly increased VOR gain during excitatory rotations in all planes compared to both the *Reference* and *Baseline only* condition. The gain improvements observed during the *Default* condition were further optimized by the other modulation types. However, increased gain improvement might even result in overfitting to the head velocities and frequencies used during testing. For example, a hyperfunctional gain of 1.5 was observed during SAN stimulation in VCI-5. It is possible that this is related to residual function [25]. A similar observation was previously reported in literature [9]. In such cases, head velocity-to-pulse amplitude sigmoidal mappings may require adjustment to reduce stimulation amplitude at these head velocities, thereby preserving therapeutic benefit while minimizing the risk of excessive VOR gain. From a clinical perspective, mild hyperfunction is likely tolerable and may decrease over time as subjects acclimatize to stimulation, as suggested by previous studies [30, 31].

Consistent with earlier findings [9], absolute gain values rarely exceeded 0.80. Based on definitions proposed for VOR gain grading, a horizontal canal VOR gain of 0.40–0.69 is considered a moderate loss and a gain of 0.70–0.79 represents mild vestibular loss [32]. Ideally, modulated vestibular stimulation should restore VOR gain ≥ 0.80. In this study, gains approaching physiological levels were achieved in three subjects (VCI-2, VCI-5, VCI-6) during excitatory impulses in the lateral and anterior planes using *Lowered baseline* or *Boosted* stimulation. Although VOR improvements were also present in the posterior canal, they did not reach values ≥ 0.70, which may relate to the lower PEV values observed during acute stimulation in this plane (Fig. 2) [10]. VCI-9 demonstrated higher PEVs during brief PAN stimulations and an increase in gain > 0.50, but this remained below 0.70.

Further analyses of saccade latencies and the temporal grouping of saccades (PR score) were beyond the scope of this study. Nevertheless, inspection of the raw traces suggested that, in conditions associated with improved VOR gain, corrective saccades occurred less frequently, with shorter latencies, and in a more organized temporal pattern. This observation is consistent with the expectation that improvements in VOR gain reduce the need for compensatory saccades, thereby providing additional support for the observed effects.

### Absence of VOR gain improvement in subgroup B

Subjects in subgroup B showed PEV values during acute LAN stimulation (block stimuli, Fig. 2) between 1 and 8 ⁰/s, which explains the absence of VOR gain improvement with modulation stimulation in this group. The subgroups highlight the heterogeneity of responses among subjects. The reduced responsiveness in subgroup B may be related to the etiology of vestibular loss. All subjects in subgroup B were diagnosed with DFNA9. However, one DFNA9 patient was included in subgroup A. The effect of this etiology, among other factors such as duration of loss, should be further investigated once larger sample sizes become available. Importantly, vestibular implant stimulation may still provide benefits by providing other forms of vestibular information to these subjects, even in the absence of a measurable VOR response.

### Limitations

Sample sizes in VCI research are currently limited due to the stepwise and complex translation of technical prototype devices into clinical studies. In this study, the exclusion of usable rotatory chair data for subjects VCI-5 and VCI-6 further reduced the statistical power. Consequently, these findings should be interpreted with caution given the exploratory nature of the current case series and will need to be confirmed in future studies involving larger cohorts as vestibular implant technology becomes more widely implemented. Second, hardware constraints resulted in unequal sampling frequencies between mid- and high-frequency VOR assessments; specifically, rotatory chair eye-movement recordings were sampled from 173 Hz instead of 250 Hz. However, the sampling frequency of 173 Hz did not result in sampling artefacts. Third, vertical canal vHIT data should be interpreted with caution, as VOR gain values from vertical vHIT in BV patients are associated with poor to moderate test–retest reliability [33]. Lastly, this study reflects the effects of four consecutive days of vestibular stimulation with one condition. It is possible that a longer intervention period would induce (greater) neural plasticity, potentially resulting in different or more pronounced effects over time.

### Implications for future VOR restoration and daily use

The findings of this study have several implications for future VOR restoration during day-to-day use of the VCI. The ability to enhance VOR gain across multiple frequencies suggests that stimulation strategies may ultimately improve dynamic visual acuity in daily life, consistent with previous laboratory work [34–36]. Next studies should focus on the functional performance in real-life conditions, by evaluating VOR responses during functional head impulse tests, dynamic visual acuity tests and patient reported outcome measures. In particular, efforts should be made to relate improvements in VOR function to the degree of perceived oscillopsia reported by patients. Recent studies quantified head motion during walking, reporting head velocities in the range of 19 to 29 ⁰/s [37, 38][*Fawden *et al*., under review*]. This implies that the measured peak head velocities in this study should fall within a functionally relevant range, suggesting sufficient VOR restoration to support effective gaze stabilization during walking. However, not all patients will experience the same amount of oscillopsia, or the same amount of effect of the vestibular stimulation. This might be related to the amount of retinal error and the degree of compensation for this problem [39].

Stimulation parameters must be carefully optimized to avoid overfitting at low head velocities, and underfitting at higher velocities within a range relevant for natural movements. Fitting strategies that were applied in this study are still investigational and can be optimized by finding the amplitude mappings that result in the best VOR restoration. For example, acceleration can be used as input, instead of or alongside velocity. Or implant design can be redesigned to have multiple electrodes in order to enable bipolar stimulation. These alternatives might improve PEV for those subjects where a limited dynamic range is observed. In other words, subgroups need other strategies in order to come to a relevant electrically evoked VOR. Moreover, mappings could be changed to increase not only the absolute VOR gain value, but also the timing of eye response related to the head impulse. Of course, if the eye response is correctly scaled but not properly timed relative to the head impulse, oscillopsia will still persist. Importantly, the inter-individual variability observed in these VOR responses indicates that fitting of vestibular implants should be performed on a personalized way. In addition, the evolution of these responses over time, including potential neuroplasticity, might be relevant in future studies. As observed in previous animal studies, PEV values change over time, suggesting the need for ongoing fitting adaptations [40]. Therefore, VOR restoration may also change accordingly. Long term studies are essential to better understand these adaptive processes.

Taken together, these results support the further use of individualized modulation strategies in long-term home use trials to optimize VOR restoration. A key next challenge will be to optimize fitting for all relevant frequency domains, thereby introducing multi-frequency modulation. An additional challenge will be to develop fitting strategies that optimize not only VOR performance but also broader functional outcomes, such as self-motion perception and balance.

## Conclusion

This study shows that motion-modulated vestibular implant stimulation can substantially enhance VOR function across mid- and high-frequency domains in patients with bilateral vestibulopathy. While excitatory responses showed robust and consistent improvements, inhibitory responses remained mostly absent. VOR gains approaching functional levels (> 0.7) were achieved in several subjects, highlighting the clinical potential of vestibular stimulation. Vestibular implant stimulation without motion-modulated stimulation did not result in VOR gain improvement. Continued refinement of stimulation conditions and individually tailored fitting approaches will be essential to further improve gaze stabilization and, ultimately, reduce oscillopsia in daily life. In addition, further research is needed to better understand variability in PEV responses across patients.

## Supplementary Information

Below is the link to the electronic supplementary material.

Supplementary file1 (DOCX 15 KB)

Supplementary file2 (DOCX 16 KB)

Supplementary file3 (DOCX 276 KB)

Supplementary file4 (DOCX 230 KB)

Supplementary file5 (DOCX 152 KB)

Supplementary file6 (DOCX 208 KB)

## Data Availability

Not yet available.
