# Supplementary material for: Vestibulocochlear implant (VCI) modulation improves mid and high frequency vestibulo-ocular reflex (VOR)
Source: J Neurol. 2026 Sep 8;273(10):576. doi: 10.1007/s00415-026-14109-0 (PMC13553661; doi:10.1007/s00415-026-14109-0)
Supplement: Supplementary file 1 [file 415_2026_14109_MOESM1_ESM.docx]

**Article title**: Vestibulocochlear implant (VCI) modulation improves mid and high frequency vestibulo-ocular reflex (VOR)
**Journal name**: Journal of Neurology
**Author names**: B.L. Vermorken, S.C.J. Van Boxel, B. Volpe, N. Guinand, A. Pérez Fornos, E. Devocht, R. van de Berg
**Affiliation, e-mail address**: Department of Otorhinolaryngology and Head and Neck Surgery, Division of Balance Disorders + Mental Health and Neuroscience Research Institute (MHeNs), Maastricht University, Maastricht, The Netherlands.
[bernd.vermorken@mumc.nl](mailto:bernd.vermorken@mumc.nl)

**Supplementary Materials I**
Individual stimulation rate per subject

| **Subject** | **100% Baseline rate (pulses per second)** |
| --- | --- |
| VCI-1 | 343 |
| VCI-2 | 400 |
| VCI-3 | 322 |
| VCI-4 | 324 |
| VCI-5 | 387 |
| VCI-6 | 354 |
| VCI-7 | 361 |
| VCI-8 | 350 |
| VCI-9 | 360 |
