# Supplementary material for: Vestibulocochlear implant (VCI) modulation improves mid and high frequency vestibulo-ocular reflex (VOR)
Source: J Neurol. 2026 Sep 8;273(10):576. doi: 10.1007/s00415-026-14109-0 (PMC13553661; doi:10.1007/s00415-026-14109-0)
Supplement: Supplementary file 2 [file 415_2026_14109_MOESM2_ESM.docx]

**Article title**: Vestibulocochlear implant (VCI) modulation improves mid and high frequency vestibulo-ocular reflex (VOR)
**Journal name**: Journal of Neurology
**Author names**: B.L. Vermorken, S.C.J. Van Boxel, B. Volpe, N. Guinand, A. Pérez Fornos, E. Devocht, R. van de Berg
**Affiliation, e-mail address**: Department of Otorhinolaryngology and Head and Neck Surgery, Division of Balance Disorders + Mental Health and Neuroscience Research Institute (MHeNs), Maastricht University, Maastricht, The Netherlands.
[bernd.vermorken@mumc.nl](mailto:bernd.vermorken@mumc.nl)

**Supplementary Materials II**
Dynamic range (DR) widths established during the first day of the first stimulation week per subject and electrode measured with 50% baseline stimulation (in current units)
LAN = lateral ampullary nerve; SAN = superior ampullary nerve; PAN = posterior ampullary nerve

| **Subject ID** | **LAN Baseline DR** | **SAN Baseline DR** | **PAN Baseline DR** |
| --- | --- | --- | --- |
| VCI-1 | 75 | 175 | 150 |
| VCI-2 | 375 | 325 | 350 |
| VCI-3 | 250 | 200 | 200 |
| VCI-4 | 325 | 350 | 400 |
| VCI-5 | 300 | 375 | 500 |
| VCI-6 | 225 | 225 | 150 |
| VCI-7 | 300 | 300 | 350 |
| VCI-8 | 475 | 575 | 875 |
| VCI-9 | 225 | 250 | 250 |
