# Supplementary material for: Vestibulocochlear implant (VCI) modulation improves mid and high frequency vestibulo-ocular reflex (VOR)
Source: J Neurol. 2026 Sep 8;273(10):576. doi: 10.1007/s00415-026-14109-0 (PMC13553661; doi:10.1007/s00415-026-14109-0)
Supplement: Supplementary file 5 [file 415_2026_14109_MOESM5_ESM.docx]

**Article title**: Vestibulocochlear implant (VCI) modulation improves mid and high frequency vestibulo-ocular reflex (VOR)
**Journal name**: Journal of Neurology
**Author names**: B.L. Vermorken, S.C.J. Van Boxel, B. Volpe, N. Guinand, A. Pérez Fornos, E. Devocht, R. van de Berg
**Affiliation, e-mail address**: Department of Otorhinolaryngology and Head and Neck Surgery, Division of Balance Disorders + Mental Health and Neuroscience Research Institute (MHeNs), Maastricht University, Maastricht, The Netherlands.
[bernd.vermorken@mumc.nl](mailto:bernd.vermorken@mumc.nl)

**Supplementary Materials V**
Representative pooled traces from the lateral, excitatory, video Head Impulse Tests for all stimulation conditions in subject VCI-1, subgroup B. (a) Reference, (b) Baseline only, (c) Default, (d) Lowered baseline, (e) Boosted. Head traces displayed in orange; eye traces displayed in blue.
